# Decomposition analysis on the equity of health examination utilization for the middle-aged and elderly people in China: based on longitudinal CHARLS data from 2011 to 2018

**DOI:** 10.1186/s12889-024-18068-x

**Published:** 2024-04-10

**Authors:** Min Su, Tianjiao Zhang, Weile Zhang, Zhengrong Li, Xiaojing Fan

**Affiliations:** 1 School of Public Administration, Inner Mongolia University, Yuquan District, Zhaojun Road, Hohhot, 010070 Inner Mongolia China; 2 School of Public Policy and Administration, Xi’an Jiaotong University, No. 28 Xianning West Road, Xi’an, 710049 Shaanxi China

**Keywords:** Middle-aged and elderly, Health examination service utilization, Health equity, Concentration index

## Abstract

**Background:**

This study aimed to investigate the utilization rate and equity of health examination service among the middle-aged and elderly population in China from 2011 to 2018. The contribution of various determinants to the inequity in health examination service utilization was also examined.

**Methods:**

Data from the China Health and Retirement Longitudinal Survey (CHARLS) were analyzed to assess the health examination service utilization rate among the middle-aged and elderly population. A concentration curve and concentration index were employed to measure the equity of health examination service utilization and decomposed into its determining factors. Horizontal inequity index was applied to evaluate the trends in equity of health examination service.

**Results:**

The health examination service utilization rates among the middle-aged and elderly population were 29.45%, 20.69%, 25.40%, and 32.05% in 2011, 2013, 2015, and 2018, respectively. The concentration indexes for health examination service utilization were 0.0080 (95% CI: − 0.0084, 0.0244), 0.0155 (95% CI: − 0.0054, 0.0363), 0.0095 (95% CI: − 0.0088, 0.0277), and − 0.0100 (95% CI: − 0.0254, 0.0054) from 2011 to 2018, respectively. The horizontal inequity index was positive from 2011 to 2018, evidencing a pro-rich inequity trend. Age, residence, education, region, and economic status were the major identified contributors influencing the equity of health examination service utilization.

**Conclusions:**

A pro-rich inequity existed in health examination service utilization among the middle-aged and elderly population in China. Reducing the wealth and regional gap, providing equal educational opportunities, and strengthening the capacity for chronic disease prevention and control are crucial for reducing the inequity in health examination service utilization.

## Background

Health examination is the most cost-effective and efficient approach for preventing and detecting diseases, reducing mortality, and improving health status [1–3]. The World Health Organization recognizes that approximately one third of diseases can be diagnosed early and treated effectively based on health examination reports, significantly impacting patient prognosis. Therefore, health examination plays a crucial role in early diagnosis, treatment, and rehabilitation [4, 5]. Experience has shown that health examinations can effectively identify healthy populations, low-risk populations, high-risk populations, and patients, allowing for timely and targeted health interventions for each. This approach helps reduce economic losses caused by diseases and decreases health inequality [6–8]. In 2009, the Chinese government initiated the National Essential Public Health Service to improve the health status of residents. This policy required the establishment of electronic health records for the middle-aged and elderly, with free health examinations included. The primary objectives of these examinations are to enhance disease screening, prevention, and health promotion, thereby controlling health costs, alleviating the health burden, and improving the health status of the middle-aged and elderly [9].

Promoting equity in the utilization of health examination service helps narrow the health gap among different populations and reduces the economic burden of health care. Equity in this utilization refers to the absence of disparities in the quantity and quality of health examination service received by individuals with the same health examination needs, regardless of their social status, income level, race, geography, or other factors [10]. This is an important measure of the performance of the public health service system and a key basis for the allocation of public health resources. In the Healthy China Action (2019–2030) plan, the Chinese government emphasized the need to improve the fairness, accessibility, and effectiveness of National Essential Public Health Service. One approach to achieving this is by providing free annual health examinations for the middle-aged and elderly, thereby promoting their health status and ensuring equity in the health examination service [11].

Research on health examination service utilization is abundant. Firstly, several studies have shown that different populations exhibit variations in the utilization rate, influenced by internal and external factors. A study in the Luton area of the UK revealed a health examination service utilization rate of 44%, with disparities observed based on age, gender, and poverty level [12]. Furthermore, researchers have employed multiple regression models to demonstrate racial disparities in such service utilization. For instance, middle-aged and elderly people of Chinese and Indian ethnicities had higher utilization rates than other middle-aged and elderly people in Malaysia [13]. A sampling survey conducted in Shandong, China, found utilization rates of 76.2% in urban areas and only 36.4% in rural areas [14]. Using a coarsened exact matching method, a study analyzed the utilization disparities of health examination service between mobile and non-mobile middle-aged and elderly populations in China. They found a utilization rate of 35.6% for the mobile group, which was lower than that of the non-mobile group [15]. Secondly, the equity of health examination service utilization has attracted significant research attention. Kypridemos et al. conducted a policy evaluation of the National Health Service health examination program in the UK, revealing absolute and relative inequalities in service utilization, favoring individuals with lower economic status [16]. Other scholars analyzed the socioeconomic inequality in health examination service utilization among Saudi Arabian residents and concluded that preventive health examination service utilization was predominantly concentrated among the more affluent population. They recommended promoting such service utilization among vulnerable groups to reduce inequality [17]. Chu et al. analyzed the utilization of health examination service among the middle-aged and elderly in Taiwan during the 2007–2008 global financial crisis and found widespread income-related inequality, favoring the higher-income population [18]. Jens et al. employed logistic regression models to analyze the factors affecting health examination service utilization among German residents. The results indicated that lower health examination service utilization was mainly concentrated among those with poorer economic status, smokers, and those who did not engage in physical activity [19]. A cross-sectional study in the UK demonstrated that factors such as gender, age, and ethnicity significantly influenced the equity of health examination service utilization among clinic attendees [20]. Scholars have also identified factors contributing to the unequal utilization of health service among the mobile middle-aged and elderly populations in China, including duration of residence, household registration, education, family economic status, and participation in health insurance [15]. These studies provide a valuable and diverse theoretical foundation for this paper. However, they have several common limitations. Firstly, most current research analyzed health examination service utilization based on cross-sectional data without assessing trends in utilization rate and equity over time. Secondly, many studies utilized logistic regression models, Poisson regression, or chi-square tests to analyze the factors influencing health examination service utilization, but they did not quantitatively analyze the factors affecting equity in health examination service utilization.

The purpose of this study was threefold. First, based on the data from the China Health and Retirement Longitudinal Study (CHARLS) for 2011 to 2018, it aimed to update information on the utilization rate of health examination service among the middle-aged and elderly population in China. Second, it used a concentration index and decomposition analysis to measure the equity of health examination service utilization among this population and assess the contributions of various factors to the observed inequity. Third, the study aimed to provide scientific evidence and recommendations for addressing health care inequalities and improving relevant policy in China.

## Methods

### Ethics

The ethics review committee of Peking University approved the CHARLS (approval number IRB00001052–11015). Informed consent was obtained, and the data were anonymized for analysis.

### Data

The data for this study were drawn from the CHARLS conducted from 2011 to 2018. This survey, led by the National School of Development at Peking University in collaboration with the China Social Science Survey Center and the Youth League Committee of Peking University, aimed to collect a high-quality set of micro-level data representing Chinese households and middle-aged and elderly individuals [21]. The data were used to analyze population aging issues in China (the data and questionnaire can be obtained through the website http://charls.pku.edu.cn/). The study focused on individuals aged 45 years and above. After data cleansing, a total of 11,496 individuals participated in the surveys conducted in 2011, 2013, 2015, and 2018. Thus, this study utilized panel data consisting of 11,496 individuals for analysis.

### Measures

#### Definition of health examination service utilization

This study determined whether respondents utilized health examination service in a given year by using the CHARLS survey question “When was your most recent routine physical examination?”.

#### Definition of other relevant variables

The economic status of the middle-aged and elderly individuals’ households was measured by dividing their self-reported household consumption expenditure minus health care expenditures into three equal groups: low, middle, and high. Research experience has shown that self-reported consumption expenditure has less bias due to deception compared to self-reported income [22]. Subtracting health care expenditures provides a more accurate reflection of the household’s actual disposable income. Need variables are the unavoidable determinants of health examination service utilization. As suggested by Wagstaff and Van Doorslaer, gender, age, self-assessed health, and functional limitation are commonly employed as indicators reflecting health need variables [23–27]. In this study, the need variables for health examination service utilization were age, gender, disability, and chronic diseases. According to the National Bureau of Statistics, China’s provinces are divided into three regions: East, Central and West. The “control variables” were education, marital status, participation in health insurance, economic status, smoking status, and alcohol consumption.

### Health inequality

The concentration index is a method recommended by the World Bank for measuring health care service utilization and health equity under socioeconomic conditions [28]. In this study, the concentration index was employed to measure the equity of health examination service utilization among middle-aged and elderly people with different socioeconomic statuses. It is calculated as twice the area between the concentration curve and the line of equality, ranging from − 1 to 1. The formula for calculating the concentration index is:

1 $${\text{C}}=\frac{2}{\mu }cov({Y}_{i},{R}_{i})$$

where “$cov$” represents the covariance, “${Y}_{i}$” denotes the variable of health examination service utilization, and “$\upmu$" represents its mean. “${R}_{i}$” represents the proportion of individuals ranked by economic level, with the ith individual being the i/N proportion of the total population. ${R}_{i}$ = i/N, where i = 1 corresponds to the individual with the lowest income and i = N corresponds to the individual with the highest income. A concentration index of 0 indicates absolute equity, and a value of 1 indicates absolute inequality. A positive concentration index suggests that individuals with higher incomes utilize more health examination service, whereas a negative value indicates that individuals with lower incomes utilize more health examination service.

### Decomposition methods of concentration index

The concentration index decomposition method was introduced by Wagstaff in 2003 [29]. Its purpose is to explore the sources of health care service utilization and health inequality. It decomposes the concentration index into contributions from influencing factors to inequity. The model for the concentration index decomposition is:

2 $${\text{y}}={\alpha }^{m}+{\sum }_{j}{\beta }_{j}^{m}{x}_{j}+{\sum }_{k}{\gamma }_{k}^{m}{Z}_{k}+\varepsilon$$

where ${\text{y}}$ represents the variable of health examination service utilization and ${x}_{j}$ represents the need variables of health examination service utilization. ${Z}_{k}$ represents the controlled variable of health examination service utilization; ${\beta }_{j}^{m}$ and ${\gamma }_{k}^{m}$ are marginal effects of each variable, that is ${dy/dx}_{j}$ and ${dy/dz}_{k}$. ε represents residual. The decomposition results of the concentration indices of the dependent variable ${\text{y}}$ are:

3 $${\text{C}}={\sum }_{j}\left({}^{{\beta }_{j}^{m}\overline{{x }_{j}}}\!\left/ \!{}_{\mu }\right.\right){C}_{j}+{\sum }_{k}({}^{{\gamma }_{k}^{m}\overline{{z }_{k}}}\!\left/ \!{}_{\mu }\right.){C}_{k}+^{{GC}_{\varepsilon }}\!\left/ \!{}_{\mu }\right.$$

${\text{C}}$ represents the concentration index of ${\text{y}}$, and $\upmu$ is the mean of ${\text{y}}$. ${C}_{j}$ and ${C}_{k}$ are the concentration indices of ${x}_{j}$ and ${z}_{k}$. ${GC}_{\mu }$ represents the concentration index of the residual term. $\overline{{x}_{j}}$ and $\overline{{z}_{k}}$ are the means of ${x}_{j}$ and ${z}_{k}$, respectively. The formula indicates that the concentration index C of health examination service utilization is the weighted sum of the concentration index of the need variables and control variables. The contribution of each need variable and control variable to inequity is calculated as the product of their concentration index and respective weight. After eliminating the contribution of the need variables to inequity, the horizontal equity can be computed.

### Analytical strategy

Categorical variables were described using percentages, and continuous variables were described using means ± standard deviations. All data were analyzed using STATA 15.0 software, and the significance level for all hypothesis tests was set at 0.05. Given that the outcome variable is binary, this study employed the Probit model to decompose the concentration index.

## Results

### Descriptive statistics

According to the descriptive statistics in Table 1, approximately 34.53% of participants came from the eastern region. The study population consisted of slightly more men than women, accounting for 52.85% and 47.15%, respectively. The majority of participants fell within the age range of 51—60 years (39.39%), and most had an educational level of elementary school or below (67.90%). Around 89.76% of respondents were married, and approximately 94.55% had health insurance coverage. The average per capita consumption expenditure of the households was 5419.10 yuan per year. Among the participants, 39.07% were smokers, 33.48% reported alcohol consumption, 4.08% had a disability, and 67.09% had chronic diseases. Statistically significant differences existed in most sociodemographic characteristics between urban and rural individuals.

**Table 1:** Descriptive characteristics of middle-aged and elderly people in China (2011,*n* = 11,496)

Variables	Total (*n* = 11,496)	Rural (*n* = 9,457)	Urban (*n* = 2,039)	*P*
Region				< 0.001
West	3,755 (32.66)	3,222 (34.07)	533 (26.14)	
Central	3,772 (32.81)	2,931 (30.99)	841 (41.25)	
East	3,969 (34.53)	3,304 (34.94)	665 (32.61)	
Sex				
Male	6,076 (52.85)	5,105 (53.98)	971 (47.62)	< 0.001
Female	5,420 (47.15)	4,352 (46.02)	1,068 (52.38)	
Age (years)				
45—50	2,640 (22.96)	2,232 (23.60)	408 (20.01)	< 0.001
51—60	4,528 (39.39)	3,774 (39.91)	754 (36.98)	
61—70	3,121 (27.15)	2,509 (26.53)	612 (30.01)	
≥ 71	1,207 (10.50)	942 (9.96)	265 (13.00)	
Education				
Elementary school and below	7,806 (67.90)	7,032 (74.36)	774 (37.96)	< 0.001
Junior high school and above	3,690 (32.10)	2,425 (25.64)	1,265 (62.04)	
Marital status				
Marriage	10,319 (89.76)	8,480 (89.67)	1,839 (90.19)	0.480
Others	1,177 (10.24)	977 (10.33)	200 (9.81)	
Insurance				
No	626 (5.45)	432 (4.57)	194 (9.51)	< 0.001
Yes	10,870 (94.55)	9,025 (95.43)	1,845 (90.49)	
Household expenditure per capita (CNY)				
Mean ± S.D	5,419.10 (5472.43)	4,803.33 (4802.00)	8,275.96 (7211.59)	< 0.001
Low	1,710.87 (722.48)	1,703.65 (722.65)	1,796.38 (716.17)	0.381
Medium	4,036.74 (763.22)	4,010.84 (762.27)	4,181.67 (752.88)	< 0.001
High	10,511.39 (6867.90)	9,900.45 (6404.24)	11,900.81 (7643.87)	< 0.001
**Life style**				
Smoke				
Yes	4,492 (39.07)	3,707 (39.20)	785 (38.50)	0.557
No	7,004 (60.39)	5,750 (60.80)	1,254 (61.50)	
Drink				
Yes	3,849 (33.48)	3,160 (33.41)	689 (33.79)	0.744
No	7,647 (66.52)	6,297 (66.59)	1,350 (66.21)	
**Health status**				
Disability				
Yes	469 (4.08)	404 (4.27)	65 (3.19)	0.025
No	11,027 (95.92)	9,053 (95.73)	1,974 (96.81)	
Chronic disease				
Yes	7,713 (67.09)	6,286 (66.47)	1,427(69.99)	< 0.002
No	3,783 (32.91)	3,171 (33.53)	612 (30.01)	

*S.D*. Standard deviation, *P* p-value.

### Status and trend of health examination service utilization

**Fig. 1:**
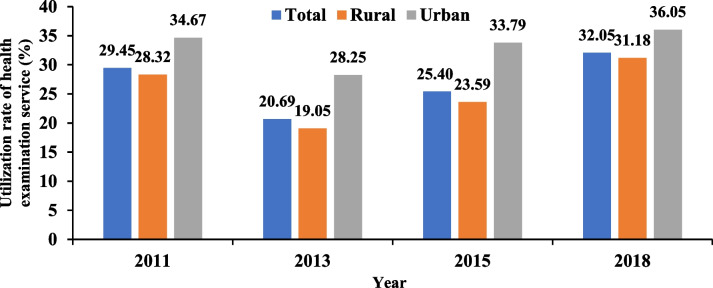
Utilization rate of health examination service for middle-aged and older people, 2011–2018

In 2011, the utilization rate of health examination service among the middle-aged and elderly was 29.45% (34.67% in urban areas and 28.32% in rural areas). In 2013, the rate decreased to 20.69% (28.25% in urban areas and 19.05% in rural areas). In 2015, the rate slightly increased to 25.40% (33.79% in urban areas and 23.59% in rural areas). In 2018, the utilization rate further rose to 32.05% (36.05% in urban areas and 31.18% in rural areas). More details could be found in Fig. 1.

### Factors associated with health examination service utilization

A multivariate logistic regression model (two-way fixed effect model) was fitted with the utilization of health examination service as the dependent variable, and the results are shown in Table 2. The results indicated that rural areas residence, year of survey, age, education, marital status, participation in health insurance, smoking status, and chronic disease were statistically associated with the utilization of health examination service (*P* < 0.05). Specifically, compared to the year 2011, the utilization rates of health examination service among the middle-aged and elderly decreased by 0.58 times and 0.77 times in 2013 and 2015, respectively, whereas it increased by 1.15 times in 2018. The utilization rate in the age groups of 51—60, 61—70, and ≥ 71 years increased by 1.29 times, 2.23 times, and 2.04 times, respectively, compared to the age group of 45—50 years. Those with at least a junior high school education had a 1.18 times higher utilization rate compared to those with a primary school education or below. The utilization rate among the middle-aged and elderly who were not married decreased by 0.91 times compared to those who were married. Participation in health insurance increased the utilization rate of health examination service by 1.25 times. The utilization rate among middle-aged and elderly individuals who smoked decreased by 0.92 times. Individuals with chronic diseases had a 1.27 times higher utilization rate of health examination service. Urban areas residence, year of survey, region, age, education, participation in health insurance, and chronic disease were statistically associated with the utilization of health examination service (*P* < 0.05).Compared to the year 2011, the utilization rates of health examination service among the middle-aged and elderly decreased by 0.74 times in 2013. Compared to the age group of 45—50 years, the health examination service utilization rates in the age groups of 61—70 and ≥ 71 years increased by 1.67 times and 1.62 times, respectively. Middle-aged and elderly individuals with at least a junior high school education had a 1.21 times higher utilization rate of health examination service compared to those with primary school education or below. Participation in health insurance increased the utilization rate of health examination service by 1.68 times. Individuals with chronic disease had a 1.33 times higher utilization rate of health examination service.

**Table 2:** Analysis of influencing factors on the utilization rate of health examination service for middle-aged and elderly people (*n* = 11,496)

**Variables**	**Group**	**Rural (*n* = 9,457)**				**Urban (*n* = 2,039)**			
		**OR**	**95%CI**		***P***	**OR**	**95%CI**		***P***
			**Lower**	**Upper**			**Lower**	**Upper**	
**Demographics**									
Time (year)	2011	1.00				1.00			
	2013	0.58	0.55	0.63	< 0.001	0.74	0.65	0.85	< 0.001
	2015	0.77	0.72	0.82	< 0.001	0.96	0.84	1.10	0.054
	2018	1.15	1.08	1.22	< 0.001	1.07	0.94	1.22	0.333
Region	West	1.00				1.00			
	Central	1.02	0.96	1.08	0.462	0.83	0.74	0.94	0.002
	East	1.03	0.98	1.09	0.265	1.11	0.98	1.25	0.102
Sex	Female	1.00				1.00			
	Male	1.06	0.99	1.13	0.118	1.07	0.94	1.22	0.327
Age (years)	45—50	1.00				1.00			
	51—60	1.29	1.21	1.38	< 0.001	1.11	0.97	1.28	0.124
	61—70	2.23	2.07	2.40	< 0.001	1.67	1.45	1.93	< 0.001
	≥ 71	2.04	1.85	2.25	< 0.001	1.62	1.36	1.95	< 0.001
Education	Elementary school and below	1.00				1.00			
	Junior high school and above	1.18	1.11	1.26	< 0.001	1.21	1.08	1.34	< 0.002
Marital status	Marital	1.00				1.00			
	Others	0.91	0.84	0.99	0.025	1.12	0.95	1.32	0.174
Insurance	No	1.00				1.00			
	Yes	1.25	1.11	1.41	< 0.001	1.68	1.40	2.02	< 0.001
Household expenditure per capita (CNY)									
	Low	1.00				1.00			
	Medium	0.98	0.93	1.04	0.516	1.01	0.87	1.19	0.865
	High	1.02	0.96	1.09	0.455	1.07	0.92	1.23	0.394
**Life style**									
Smoke	No	1.00				1.00			
	Yes	0.92	0.86	0.98	0.014	0.90	0.79	1.03	0.116
Drink	No	1.00				1.00			
	Yes	0.96	0.90	1.01	0.123	1.04	0.93	1.17	0.475
**Health status**									
Disability	No	1.00				1.00			
	Yes	0.94	0.84	1.06	0.317	0.78	0.59	1.04	0.090
Chronic disease	No	1.00				1.00			
	Yes	1.27	1.20	1.33	< 0.001	1.33	1.20	1.49	< 0.001

*OR* Odds ratio, *95%CI* 95% confidence interval.

### Equity in the health examination service utilization

**Fig. 2:**
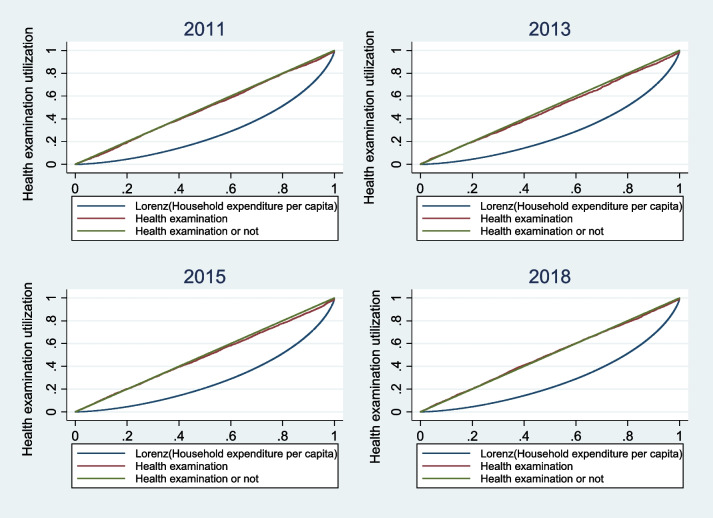
The concentration curves of health examination service utilization from 2011 to 2018

Figure 2 presents the concentration curves of health examination service utilization among the middle-aged and elderly from 2011 to 2018. The concentration curves for this period are consistently below the line of equality, indicating a greater concentration of utilization among individuals with higher economic status. The concentration index for health examination service utilization among middle-aged and elderly people in 2011, 2013, 2015, and 2018 were 0.0080, 0.0155, 0.0095, and − 0.0100, respectively. The horizontal inequity indices were 0.0130, 0.0467, 0.0385, and 0.0254, respectively. Both the concentration index and horizontal inequity indices were positive, indicating that health examination service utilization was mainly concentrated among middle-aged and elderly people with higher economic status. In rural areas, the concentration indices for health examination service utilization in 2011, 2013, 2015, and 2018 were − 0.0060, − 0.0135, − 0.0236, and − 0.0352, respectively. The horizontal inequity indices were − 0.0010, 0.0177, 0.0054, and 0.0002, respectively. The negative values of the concentration index indicated that health examination service utilization was mainly concentrated among middle-aged and elderly people with lower economic status. However, according to the definition of the horizontal inequity index, this could only be considered unfair after removing the influence of the need factor from the concentration index. Except for 2011, the horizontal inequity indices in all years were positive, indicating that health examination service utilization was still mainly focused on middle-aged and elderly people with higher economic status. In urban areas, the concentration indices for health examination service utilization in 2011, 2013, 2015, and 2018 were 0.0027, − 0.0036, 0.0170, and 0.0546, respectively. The horizontal inequity indices were 0.0077, 0.0276, 0.0460, and 0.0900, respectively. Both the concentration indices and horizontal inequity indices were positive, indicating that health examination service utilization was mainly concentrated among middle-aged and elderly people with higher economic status (Table 3).

**Table 3:** Concentration index and horizontal equity in the health examination service utilization among the elderly from 2011 to 2018

Time	Total (*n* = 11,496)				Rural (*n* = 9,457)				Urban (*n *= 2,039)			
	**CI**	**95% CI**		**HI**	**CI**	**95% CI**		**HI**	**CI**	**95% CI**		**HI**
		**Lower**	**Upper**			**Lower**	**Upper**			**Lower**	**Upper**	
2011	0.0080	− 0.0084	0.0244	0.0130	− 0.0060	− 0.0246	0.0125	− 0.0010	0.0027	− 0.0319	0.0374	0.0077
2013	0.0155	− 0.0054	0.0363	0.0467	− 0.0135	− 0.0377	0.0107	0.0177	− 0.0036	− 0.0437	0.0366	0.0276
2015	0.0095	− 0.0088	0.0277	0.0385	− 0.0236	− 0.0447	− 0.0025	0.0054	0.0170	− 0.0184	0.0524	0.0460
2018	− 0.0100	− 0.0254	0.0054	0.0254	− 0.0352	− 0.0525	− 0.0178	0.0002	0.0546	0.0209	0.0883	0.0900

*CI* Concentration index, *95%CI* 95% confidence interval, *HI* Horizontal inequity index.

### Contribution to equity in the health examination service utilization

A Probit model was established in the study. Based on the fitted model, further decomposition of the concentration index was conducted to determine the contribution rates of various factors to the inequity in health examination service utilization. When the contribution rate is positive, this indicates that the factor increases the inequity favoring the wealthier individuals (those with higher economic status utilizing more health examination service). Conversely, for individuals with lower economic status, it implies a higher utilization of the health examination service. Table 4 presents the decomposition results of the concentration index for health examination service utilization among the middle-aged and elderly from 2011 to 2018. In 2011, rural area residence made the largest contribution to the inequity (132.65%), followed by education (54.25%), age (− 51.96%), and region (− 38.11%). In 2013, age made the greatest contribution (197.69%), followed by rural areas residence (117.97%), education (84.76%), and economic status (81.84%). In 2015, age had the largest contribution to the inequity (− 299.27%), followed by rural areas residence (186.29%), education (145.80%), and region (34.86%). In 2018, age was the largest contributor to the inequity (355.66%), followed by rural areas residence (− 67.30%), education (− 45.51%) and economic status (− 45.69%).

**Table 4:** Decomposition of concentration index for the health examination service utilization among the elderly from 2011 to 2018

Variables	2011		2013		2015		2018	
	Contribution to CI	Contribution (%)	Contribution to CI	Contribution (%)	Contribution to CI	Contribution (%)	Contribution to CI	Contribution (%)
Residence (rural)	0.0106	132.65	0.01825	117.97	0.0177	186.29	0.0067	−67.30
Central	−0.0005	−6.16	−0.0004	−2.33	0.0000	0.63	0.0010	−9.54
East	−0.0025	−31.95	0.0026	17.03	0.0032	34.23	−0.0003	3.09
Male	−0.0008	−9.40	−0.0002	−1.14	−0.0006	−6.17	−0.0002	2.00
51—60 (year)	−0.0000	−0.18	−0.0008	−4.94	−0.0005	−5.35	−0.0021	20.72
61—70 (year)	−0.0028	−34.56	−0.0145	−93.90	−0.0171	−180.29	−0.0215	216.10
≥71(year)	−0.0014	−17.22	−0.0153	−98.85	−0.0108	−113.63	−0.0119	118.84
Junior high school and above	0.0043	54.25	0.0131	84.76	0.0138	145.80	0.0046	−45.51
Marital status (others)	0.0006	0.76	−0.0001	−0.47	0.0002	1.87	0.0008	−8.06
Insurance (Yes)	0.0002	2.45	0.0002	0.98	0.0002	2.12	0.0003	−2.71
Medium (CNY)	−0.0000	−0.01	0.0000	0.01	−0.0000	−0.02	−0.0000	0.01
High (CNY)	−0.0009	−11.64	0.0127	81.85	−0.0019	−20.54	0.0046	−45.70
Smoke (Yes)	−0.0001	−0.85	−0.0006	−3.72	−0.0004	−4.02	−0.0009	9.03
Drink (Yes)	−0.0021	−26.29	−0.0015	−9.58	0.0005	4.92	0.0014	−13.59
Disability (Yes)	0.0001	1.85	−0.0001	−0.71	0.0002	2.31	0.0004	−3.94
Chronic disease (Yes)	−0.0001	−1.63	−0.0003	−2.02	−0.0002	−1.94	−0.0001	1.10

## Discussion

This study updates the knowledge on the trend and equity of health examination service utilization for the middle-aged and elderly in China in two ways. First, we used the large, nationally representative longitudinal survey data in CHARLS to evaluate the level of health examination utilization from 2011 to 2018. The findings are thus more generalizable to a wider population in China and might help suggest a more convincing trend in health examination utilization. Second, this study conducted a detailed decomposition analysis of the concentration index for health examination utilization from 2011 to 2018, facilitating the identification of an effective way to reduce the inequity.

The first major finding was the analysis of health examination service utilization among the middle-aged and elderly. The overall utilization rate of health examination service among this group in China showed a slight upward trend, with urban–rural disparities. This trend can be attributed to the successful promotion and implementation of the national essential public health service. In line with the stipulations for health management service for the elderly outlined in the National Essential Public Health Service, primary healthcare institutions (such as community and township health centers) are required to conduct an annual health examination for elderly residents within their jurisdiction. This aims to facilitate their timely and effective follow-up efforts in health management and health guidance. Furthermore, the management of chronic diseases such as hypertension and diabetes has been incorporated as a crucial component [30, 31], enhancing the accessibility of health examination service for the middle-aged and elderly people. According to data from the Health Management Blue Book: Report on the Development of Health Management and Health Industry in China No. 5 (2022), the utilization rate of health examination in China increased from 25.47% in 2011 to 30.52% in 2020 [32], demonstrating a gradual increase trend consistent with the findings of this study. However, the increase in health examination service utilization among the middle-aged and elderly in China from 2011 to 2018 was relatively small. This has several potential reasons. Firstly, the concept of treating diseases rather than prevention has not changed among the middle-aged and elderly, and the shift from a disease-centered approach to a health-centered approach has not been fully realized. Therefore, the middle-aged and elderly people still lack an emphasis on health examination. Secondly, the high expenses and challenges in accessing healthcare create barriers for middle-aged and elderly individuals in China [33], hindering their utilization of health examination services and potentially resulting in a decline in the service utilization rate. Finally, the study results showed that the utilization rate of health examination service among the urban middle-aged and elderly was higher than that among the rural middle-aged and elderly, which is consistent with previous research [14, 34]. The disparities in health examination service utilization between the urban and rural middle-aged and elderly population can be attributed to unequal allocation of health resources between urban and rural areas, income disparities, and differences in mindsets and beliefs.

The second main finding was the factors influencing health examination service utilization among the middle-aged and elderly. Age, education level, participation in health insurance, chronic disease, and urban–rural disparities were the main influencing factors. As individuals age, they may pay more attention to their health conditions. With a stronger awareness of health management, they are more likely to undergo health examinations. Higher educational attainment is associated with a greater focus on personal health. Individuals with higher education tend to prioritize disease prevention, leading to higher utilization of health examination service. Furthermore, the study found that middle-aged and elderly people who participate in health insurance had a significantly higher utilization rate of health examination service compared to those without insurance, aligning with previous research on the positive impact of health insurance coverage on health care service utilization [35–37]. The increasing number of chronic diseases also contributes to the utilization of health examination service to some extent, likely due to the nature of the diseases themselves. Chronic diseases pose serious health risks, tend to worsen over time, and are prone to comorbidities. Their treatment requires long-term adherence, resulting in sustained increases in health care expenditures [38–40]. Based on these factors, individuals with chronic diseases are more likely to prioritize their health management and undergo health examinations. Lastly, urban–rural disparities are also an important influencing factor, which may be related to income disparities, healthcare infrastructure, and the accessibility of health care service between urban and rural areas. Considering the combined effects of these factors, we realize the significance of using modern statistical analysis techniques to scientifically identify vulnerable populations and implement targeted interventions. This approach is crucial for improving the utilization of health examination service, strengthening a preventive approach, and achieving universal health for all.

The third main finding focuses on the analysis of equity in health examination service utilization among this population. The concentration index showed a general decreasing trend from 2011 to 2018, and it was negative in 2018, indicating a reduction in the inequity of health examination service. This can be attributed to the improvement in economic and geographical accessibility brought about by the National Essential Public Health Service, thereby reducing the disparity in the utilization of health examination service for the middle-aged and elderly, particularly in rural areas. However, the concentration curve was below the line of equality, and the horizontal inequity index were positive. These findings suggest that health examination service utilization among the middle-aged and elderly in China was primarily concentrated among those with higher economic status. Age, residence, education, region, and economic status were identified as major factors influencing the equity of health examination service utilization among the middle-aged and elderly population, in addition to economic status. From the perspective of social welfare, this inequality favoring individuals with higher economic status poses a great challenge to disease prevention, control, and health management in China. Further decomposition of the concentration index for the utilization of health examination service among the middle-aged and elderly population revealed a positive value for the level-based inequity index, indicating that the utilization was primarily concentrated among those with higher economic status. Furthermore, by comparing and analyzing the level-based inequity index of health examination service utilization between urban and rural areas from 2011 to 2018, the study found that the disparities in the level-based inequity index between urban and rural areas were 0.0087, 0.0099, 0.0406, and 0.0898 respectively. These disparities displayed an overall upward trend, indicating that the urban–rural gap in the utilization of health examination service continues to widen, with this inequity becoming increasingly prominent. Most variables such as rural areas residence, education, marital status, participation in health insurance, and the natural logarithm of per capita consumption expenditure had positive contributions to the concentration index, thereby increasing the inequity favoring individuals with higher economic status. Consistent with previous research findings, age and chronic diseases negatively contribute to inequity, reducing the inequity favoring individuals with higher economic status [41–43]. This is because as age increases and the risk of chronic diseases rises, this segment of the middle-aged and elderly population begins to value health management and is more likely to utilize health examination service [44].

### Strengths and limitations

This study has several strengths. Firstly, the results can comprehensively represent the utilization of health examination service among the middle-aged and elderly in China. The study utilized data from the CHARLS, which covers 28 provinces, 150 county-level units, and 450 village-level units in China. The data has a large sample size, and the questionnaire content is detailed and comprehensive, ensuring the representativeness of the research results. Secondly, this study analyzed the current status and equity of health examination service utilization, not only updating the information on utilization but also allowing for a more intuitive analysis of the trends in utilization and equity of health examination service. Lastly, this study employed the concentration index and concentration index decomposition methods to comprehensively quantify the factors influencing the utilization of health examination service among the middle-aged and elderly in China. This provides a theoretical basis for targeted improvements in China’s healthcare policy.

The study also has several limitations. First, all the CHARLS data were collected via a self-reporting approach and, as such, recall bias may exist. Additionally, the availability of measured determinants of health examination service utilization was limited by the pre-specified questions in the survey, and some unobserved confounding factors may exist for which we did not control. Furthermore, although this analysis covered health examination service utilization in 2011, 2013, 2015, and 2018, it was not continuous; hence, the data may not have determined the trends in health examination service utilization and changes in equity. As continuous CHARLS data are added in the future, reexamining the trend will be important.

## Conclusions

The overall utilization rate of health examination service among middle-aged and elderly individuals in China displayed an upward trend from 2011 to 2018. However, inequity existed, with a particular focus on individuals with lower economic status. In addition to economic status, factors such as age, residence, education, and region contributed significantly to the inequities in utilization. Therefore, it is necessary to develop healthcare policies that allocate resources and service, reduce wealth and regional disparities, provide equal educational opportunities, and strengthen the prevention and control of chronic disease. These measures can help reduce the socioeconomic disparities in health examination service utilization among middle-aged and elderly population.

## Data Availability

The datasets and questionnaire are available at https://charls.charlsdata.com/pages/data/111/zh-cn.html.

## Abbreviations

CHARLS: China Health and Retirement Longitudinal Studies
OR: Odds Ratio
95% CI: 95% Confidence Interval
CI: Concentration Index
HI: Horizontal Inequity Index
